# The concept of vulnerability in medical ethics and philosophy

**DOI:** 10.1186/s13010-019-0075-6

**Published:** 2019-04-11

**Authors:** Joachim Boldt

**Affiliations:** Department of Medical Ethics and the History of Medicine, University of Freiburg, Freiburg, Germany

**Keywords:** Vulnerability, Healthcare, Ethics, Philosophy, Clinical research, Phenomenology, Hermeneutics

## Abstract

**Background:**

Healthcare is permeated by phenomena of vulnerability and their ethical significance. Nonetheless, application of this concept in healthcare ethics today is largely confined to clinical research. Approaches that further elaborate the concept in order to make it suitable for healthcare as a whole thus deserve renewed attention.

**Methods:**

Conceptual analysis.

**Results:**

Taking up the task to make the concept of vulnerability suitable for healthcare ethics as a whole involves two challenges. Firstly, starting from the concept as it used in research ethics, a more detailed characterization and systematization of the different realms of human abilities and the various ways in which these realms contain vulnerability is to be established. Secondly, at the same time, the sought-after concept of vulnerability should avoid picturing the relation between healthcare recipient and provider as a relation between a dependent individual in need and another individual capable of providing all the help necessary. An adequate concept of vulnerability should enable one to understand when and in which respects care providers may be vulnerable as well. Philosophical accounts of vulnerability can help to meet both of these challenges.

**Conclusions:**

Philosophical accounts of vulnerability can help to make the concept of vulnerability suitable for healthcare ethics as a whole. They come with a price, though. While the ethical role of vulnerability in medical ethics usually is to signify states of affairs that are to be diminished or overcome, philosophical accounts introduce forms of vulnerability that are regarded as valuable. Further analyzing and systematizing forms and degrees of vulnerability thus comprises the task to distinguish between amounts and types of vulnerability that can count as valuable, and amounts and types of vulnerability that are to be alleviated.

## Background

Vulnerability is an all-pervasive phenomenon in healthcare. Some patients’ lives depend on highly technologized care, others are confronted with severe diagnoses and a prospect of a future life with chronic disease, still others face therapies that promise cure but may cause serious adverse effects as well. And all of them, if they are in-house patients, have to adjust to an unknown and anonymous hospital environment and a daily schedule that is not set by themselves, but the healthcare team and organizational and institutional demands. The vulnerability of these patients and its different forms and levels, influences their actions, emotions, thoughts, and convictions, and it influences the actions and reactions of healthcare professionals. If one makes use of a categorization of vulnerability into physical, emotional, and cognitive, it can be argued that all these forms are found in healthcare. Patients suffer from diseases, which constitutes physical vulnerability, they experience emotional distress, and their situation often comprises cognitive uncertainty.

To address vulnerability, its forms, and its ethical significance, then, is to address one of the most central characteristics of providing and receiving care in healthcare. Given this observation, it must come as a surprise that the concept of vulnerability does not play a major role in medical ethics. It has found its main niche in medical research ethics where it is used to denote specific individuals or groups of individuals who share features that put them at an elevated risk of being swayed into consenting to take part in research. These groups and individuals thus need extra protection and assistance.

In the following, it is argued that the concept of vulnerability could enrich medical ethics beyond its application in clinical research ethics. Some approaches in medical ethics attempt to do just this, and they deserve renewed attention and further elaboration. These attempts have to meet two main conditions. Firstly, starting from the concept of vulnerability as it used in research ethics, a more detailed characterization and systematization of the different realms of human abilities and the various ways in which these realms contain vulnerability is to be established. Secondly, at the same time, the sought-after concept of vulnerability should avoid picturing the relation between healthcare recipient and provider as a relation between a dependent and needy individual, on the one hand, and another individual capable of providing all the help necessary, on the other hand. An adequate concept of vulnerability should enable one to understand when and in which respects care providers may be vulnerable as well.

In order to get a grasp on the broad significance of vulnerability and on the ways in which all human existence is characterized by vulnerability, a good place to start is philosophy. Especially theories from within the phenomenological and hermeneutic traditions introduce the concept. Phenomenological accounts can supply an analysis of physical and corresponding emotional vulnerability, hermeneutic approaches can supply an account of cognitive vulnerability and, again, its emotional counterpart. Identifying these vulnerabilities can contribute to a full understanding of the situation of healthcare recipients and its ethical implications. What is more, hermeneutic approaches can also help to explain in which ways healthcare providers, too, are marked by vulnerability.

Connecting these philosophical perspectives to medical ethics is challenging, though, since the content and use of vulnerability in each field differs significantly. While in medical ethics vulnerability is conceived of as a feature of specific groups that triggers protection and interventions to reduce vulnerability, in philosophy vulnerability is often understood as a universal feature of the human condition. This feature must be accepted as inevitable, it is assumed, and, indeed, it may even be cherished as valuable. Further analyzing and systematizing forms and degrees of vulnerability along these lines thus comprises the task to distinguish between amounts and types of vulnerability that can count as valuable, and amounts and types of vulnerability that are to be alleviated.

## Methods

This study is an exercise in ethical and philosophical reasoning. Its method is conceptual analysis.

## Results and discussion

### Vulnerability in the dictionary: physical, emotional, cognitive

Being vulnerable means, as Merriam Webster defines, being capable of becoming “physically or emotionally wounded” [1]. Illness and disease prove our physical vulnerability, and so do cuts and bruises. Likewise, if one follows Merriam Webster, grief and despair can be understood as states of being emotionally wounded that prove a prior hidden susceptibility for emotional harm. Vulnerability thus refers to a state of physical and emotional well-being that is in danger of being disturbed and destroyed due to being susceptible to harmful influences. In addition, being vulnerable also means, Merriam Webster continues, “being assailable”. If one applies the above explanation to this example, vulnerability can refer to sets of convictions and claims that are open to being criticized or rendered implausible. This introduces a third realm of vulnerability. Besides physical and emotional vulnerability there is, if one follows the dictionary, a kind of vulnerability that refers to cognitive phenomena: “cognitive vulnerability”, as one may say. Having to endure warranted criticism and having to give up and change one’s convictions accordingly brings to light an inherent prior vulnerability of one’s beliefs and convictions. What is at stake here is the assumption that convictions that one adheres to and that guide one’s behavior are correct. This assumption may become doubtful in the light of, for instance, counterexamples, new experiences, or alternative interpretations of the meaning of key terms of those convictions.

If one were to include all these three realms of human vulnerability in one definition, it becomes obvious that the language of well-being and harm that is appropriate in the case of physical and emotional vulnerability does not readily fit the case of cognitive vulnerability. If one has to give up beliefs because one is confronted with arguments or evidence that these beliefs must be false, the “harm” that is done is at the same time a “good” since the force that destroys one’s beliefs is not external but acts with an authority that one has accepted already when forming one’s original beliefs. Nonetheless, there is an element of irritation in meeting justified criticism, an element of disturbed certainty, which parallels “being wounded” in the case of physical and emotional vulnerability. The following general explication of vulnerability is meant to capture this parallel and cover all three realms of human vulnerability: Vulnerability refers to a state of physical, emotional, and cognitive stability that is in danger of being disturbed or destroyed due to being susceptible to destabilizing influences. These three dimensions of vulnerability roughly coincide with the three main domains of human abilities as introduced in developmental psychology, namely physical, social, and intellectual abilities [2].

### Vulnerability in healthcare

In medicine and healthcare, vulnerability as defined above is all-pervasive. Disease and illness, the raison d’etre of healthcare, are visible signs of the physical vulnerability of the human body. At the same time, the ill body is characterized by increased physical vulnerability itself. Compared to the healthy body its susceptibility to harm, to infections, pain, and muscle loss, e.g., is often significantly elevated. Someone who suffers from a disease is likely to experience further physical decline if no medical help is provided. Ill health thus is a factor that increases the “relative risk or susceptibility to adverse health outcomes” [3] and constitutes increased physical vulnerability.

In addition, the physical vulnerability of patients is in many cases accompanied by emotional vulnerability. The diagnosis of a severe progressive chronic condition or the prospect of death can cause fear, anger, and despair. Relatives and friends of the patient may experience grief and anxiety. Hospital architecture and clinical routines can evoke intimidation and discomfiture. Again these emotional states are, on the one hand, visible signs of a prior emotional vulnerability. On the other hand, these emotional states sometimes increase the likelihood of further harm. For example, discomfort may result in unwillingness to follow a treatment regime, being agitated negatively influences the success of therapies, and prolonged grief may result in the loss of social relations and seclusion.

Finally, in cases of severe disease, patients may exhibit and experience elevated forms of cognitive vulnerability. First of all, when one gets to know a severe diagnosis, understanding medical facts concerning this diagnosis, one’s prognosis, and treatment options is challenging, and it is challenging to grasp the impact of these facts for one’s daily routines, working life, and other activities. In this regard, cognitive vulnerability describes the elevated difficulties of patients in certain situations to grasp medical facts and facts concerning one’s life-world. What is more, grasping these facts always also incorporates evaluative aspects. When one understands the potential impact of a chronic disease on one’s future life, different scenarios are accompanied by different evaluative colorings depending on how the scenarios affect one’s interests, preferences, and ideas of what constitutes a good life. When asked to choose a treatment option, patients are supposed to examine these options from the point of view of their preferences. Again, given that these options potentially concern the rest of one’s lifetime and affect very basic preferences and values, this decision-making can be unsettling. At times, adapting one’s life to a chronic disease may call into question values and preferences that have hitherto shaped the life of a patient. Patients may feel urged to restructure and perhaps replace values they used to adhere to. The German idiomatic expression “Hauptsache gesund!”, (“Don’t worry, at least you are still healthy!”), which is used to console someone in cases of misfortune other than illness, bears witness to the high degree of priority one often assigns to health as compared to other valuable aims and states such as close social relations or a meaningful job. Patients with severe diseases may feel the need to reassess the ordering of preferences they have adhered to so far. This may result in a new order of preferences, or new interpretations of the meaning of “health”.

All this is to say that cognitive vulnerability comprises both factual understanding and evaluative reasoning. Factual understanding may be compromised by uncertainty and fear regarding one’s life to come. Evaluative reasoning involves making decisions with serious and long-term effects regarding one’s health and one’s future life, and these high stakes may cause anxiety. Evaluative reasoning may also consist of having to restructure basic values and ideas of a good life that partially define who one is and what one stands for as a person. In this case, evaluative reasoning becomes a process of personal identity transition that is oriented by experiences, preferences, and values, but that is not firmly guided by those preferences and values. Again, this process will often be accompanied by anxious uncertainty.

It is the role and task of physicians and health care providers to supply help in cases of disease. In doing so, they reduce and alleviate a patient’s physical and related emotional vulnerability. What is more, when physicians explain medical facts, and when they make an effort to ensure that the patient understand these facts by, for example, explaining repeatedly and avoiding professional jargon, they alleviate parts of the patient’s cognitive vulnerability as well. However, a first limit of the expert role is reached when it comes to applying statistic knowledge about the progress of a disease to the prospects of an individual patient. General medical knowledge concerning the progress of a disease needs to be combined with clinical data and clinical experience in order to allow predictions in individual cases. Such a prediction often resembles an educated guess more than safe knowledge. In this sense, medical knowledge is ambiguous and physicians are cognitively vulnerable [4].

Even more so, if a patient’s question, “What should I do?”, transgresses the boundaries of factual medical knowledge and becomes a matter of evaluative reasoning, invoking expert knowledge of physicians and healthcare professionals does not help to supply answers. If the patient struggles to judge future scenarios from the point of view of his preferences and values, these preferences are the decisive and safe ground to find answers. The role of the physician in this kind of conversation may thus be to assist the patient in bringing to mind and clarifying his own preferences. Finally, if the patient struggles to restructure values and to develop novel conceptions of what a good life may consist of, neither factual medical knowledge, nor knowledge of patient preferences can serve as a basis for finding conclusive answers. If the physician accompanies a patient in this process of renegotiating basic values and personal identity, both share the same kind of cognitive vulnerability. Both the patient and the physician may know stories of other patients who for different reasons and in different ways did find or did not find value in living with a certain serious disease. Which of these reasons one finds convincing and which of these reasons one becomes able to integrate into one’s self-understanding remains an individual affair, both for the physician, who may hold convictions regarding these reasons and values, and for the patient, who struggles to reposition herself.

As becomes apparent, the concept of vulnerability captures a number of states of affairs that are characteristic of the situation of patients, and partly also of professionals, in the healthcare system. These characteristics are of ethical significance. If one recognizes elevated risks of becoming physically wounded, medical assistance is called for. Emotional and cognitive vulnerability call for adequate forms of care as well. The concept of vulnerability thus not only allows identification of important features of the situation of patients in healthcare, at the same time it pinpoints potential realms where patients may need assistance. This includes medical assistance, but is not restricted to it, as visualized by cases of emotional and cognitive vulnerability.

In addition, the concept of vulnerability highlights the intimate connection between physical, emotional, and cognitive phenomena. From the perspective of the patient, experiencing symptoms of what may be a disease is unsettling, both because of the symptoms themselves which may be painful or disabling, but even more so because of the possibility that what one often regards as a naturally given prerequisite of one’s life, i.e. one’s health, is called into question. Getting to know the diagnosis may increase emotional and cognitive vulnerability. Moreover, grief induced by experiences of loss, maybe accompanied by a conviction that one cannot trust in relationships anymore, can lead to elevated physical vulnerability and illness. Since the concept of vulnerability covers all these three realms of disturbance and discomfiture, it encourages a comprehensive understanding of the patient’s situation. When a patient is diagnosed with a disease, the concept of vulnerability leads attention to possible emotional and cognitive challenges as well instead of exclusively focusing on physical needs.

### Medical research ethics: vulnerability as impairing the capacity to consent

Given the broad scope of the concept of vulnerability as described above and the potential ethical impact it may have on care in healthcare, it may come as a surprise that the concept does not play a major role in medical ethics today. Admittedly, there are classic approaches to medical ethics and philosophy of medicine that often implicitly describe the situation of patients seeking medical help as a situation of vulnerability [5]. One may argue that it is also part of the principles of beneficence and non-maleficence in the principles approach to medical ethics [6]. Nonetheless, if one focusses on explicit uses of the term, application of the concept of vulnerability in medical ethics is mainly confined to research ethics. Here, following the ethical rationale that even if information and consent procedures are in place, some groups of research participants may need extra protection, vulnerability is introduced as a supplement to informed consent. Certain groups of patients or healthy volunteers may be inclined to consent to participate in research due to factors that unduly influence their decision-making. Examples include persons at the lower end of strictly hierarchically ordered institutions (e.g. prisons, the army) who are asked to participate in research via this institution, and socially disadvantaged persons who may take part in research comprising high risk of harm because of monetary gratification, or in order to obtain otherwise inaccessible medical treatment. In the same vein, persons whose capacity to consent is impaired, inexistent, or not fully developed yet, such as the mentally impaired, advanced stage dementia patients, the comatose, and children, are termed “vulnerable” as well [7].

This consent oriented understanding of vulnerability does not ask which realm of human abilities (e.g. physical, emotional, cognitive) might be susceptible to harm, but assesses effects of the situation and features of potential research participants on their ability to provide informed consent. The situations and features may include, as mentioned, institutional membership, social status, financial background, and physical impairments of cognitive abilities. In other words, on this account situations and features of vulnerability are not systematically ordered but comprise all sorts of factors which, in turn, may contribute in different ways to doubtful consent.

This omission does not preclude the successful application of the concept in research ethics, as is apparent from its use in corresponding legislation and regulation. Nonetheless, it renders the transfer of the concept to the wider field of medical and healthcare ethics difficult. In these wider contexts, informed consent is important, too, but it is not the only ethically relevant issue. If patients are intimidated by hospital routines or are frightened by the prospect of a severe diagnosis, this may also have an effect on what they regard to be the right thing to do, but it will only seldom do so to such an extent that they would be considered not fully able or unable to consent. Vulnerability in these cases thus is not in the first place a matter of consent, but of, generally speaking, well-being.

As a reaction to this shortcoming, a number of approaches have been proposed to refine the concept of vulnerability. These approaches include proposals to assign layers of vulnerability to individuals rather than to label specific groups as vulnerable [8], to scrutinize clinical encounters and experiences of vulnerability [9], to develop a needs based instead of a consent oriented model of vulnerability [10], to embrace both notions of being harmed and being wronged within the concept of vulnerability [11], and to devise a notion of universal human vulnerability that allows to develop just institutions [12]. Given the significance of the concept of vulnerability for situations of receiving and providing healthcare, and for the ethical implications of these situations, these approaches deserve renewed attention and further elaboration.

### An ethical caveat and the twofold task of further developing the concept of vulnerability

Turning the concept of vulnerability into a core ethical concept in healthcare ethics is in danger of cementing an inappropriate asymmetry of the relation between healthcare recipient and provider. If vulnerability is sought on the side of the patient alone, and if the care provider is the one to protect and assist if vulnerability is detected, the resulting relation is constituted by the asymmetry of one individual being weak and in need and another individual being strong enough to act as aide. At times, regarding, for example, acute and treatable conditions, this asymmetry may accurately depict the situation and the ethical implications of the respective role ascriptions may not be worrisome. Nonetheless, as a general outlook, this understanding underestimates resources and knowledge of patients and it overestimates the ability of healthcare providers to supply all the help needed. Patients suffering from chronic diseases may know more about variations of their disease and its effects on everyday life than physicians, medical knowledge may be inchoate and refer to statistical regularities rather than the disease process in one individual patient, healthcare providers may be influenced by prejudices regarding certain groups of patients and thus fail to provide adequate assistance. Finally, as mentioned above, healthcare providers’ abilities to supply answers to a patient’s questions of identifying relevant preferences with regard to treatment options and of restructuring preferences and values are limited. Vulnerability as a core ethical principle may thus appear to be overly protective and not to do justice both to the situation of healthcare recipients and providers. Strengthening the concept of vulnerability thus may appear to neglect important advances in medical ethics such as the ideal of shared decision-making, empowerment, and health promotion.

Now, whether this criticism is convincing depends on the concept of vulnerability one takes as a basis. In the consent-oriented model favored by research ethics, for example, ethical actions include provisions to inform children in ways that are adequate to their age. In this case, then, to identify vulnerability does not lead to ethical provisions that lock potential research participants into a passive role but rather implies actions that diminish dependency. Thus, as long as one uses a concept of vulnerability that includes a sensitivity for factors that reduce the ability to understand and reflect, taking this concept as a basis for ethical actions does open up opportunities to alleviate asymmetry. In addition, situations in which healthcare providers are hard-pressed to live up to their supposed role as advisors can be described in terms of vulnerability as well. Most importantly, as was argued above, being challenged to cope with a severe diagnosis, and to restructure one’s preferences and values, constitutes a case of cognitive vulnerability. This form of vulnerability characterizes both the situation of healthcare recipients and providers. Being aware of this form of vulnerability thus does not highlight dependency and passivity of patients, on one side, and healthcare professionals’ knowledge and know-how, on the other side. It rather emphasizes equality of having to settle for a conception of the good life on shaking grounds.

Given these considerations, a twofold task emerges for further elaborating the concept of vulnerability in healthcare ethics. Firstly, starting from the concept as it used in research ethics, a more detailed characterization and systematization is needed of the different realms of human abilities and the various ways in which these realms contain vulnerability. Such a concept could cover many and significant ethically relevant characteristics of providing and receiving healthcare, as the above first look at healthcare from the perspective of the threefold definition of vulnerability bears witness to. Secondly, at the same time, the sought-after concept of vulnerability should avoid picturing the relation between healthcare recipient and provider as a relation between a dependent individual in need and another individual capable of providing all the help necessary. An adequate concept of vulnerability should enable one to understand when and in which respects care providers may be vulnerable as well. Especially phenomena of cognitive vulnerability are cases in point here.

### Vulnerability in philosophy

Philosophical accounts of vulnerability can be found in the phenomenological and hermeneutic traditions. In phenomenology, the concept is closely tied to an analysis of the role of the body in perception and knowledge. On these accounts, the body is not perceived of as a mere tool for perception and knowledge acquisition. Instead, the emphasis is on the ways in which the body constitutes how one relates to the world and acts in it, and on how this influence is experienced and shapes one’s emotions, perceptions, and actions. The vulnerability of the body and its needs are thus considered to have an effect on emotion, perception and knowledge, and perception and knowledge are thought of as “embodied” phenomena. Accordingly, embodiment emphasizes physical vulnerability and analyzes the traces of this vulnerability into the realm of emotion and perception. Fineman writes: “Our embodied humanity carries with it the ever-constant possibility of dependency as a result of disease, epidemics, resistant viruses, or other biologically-based catastrophes. Our bodies are also vulnerable to other forces in our physical environment: There is the constant possibility that we can be injured and undone by errant weather systems, such as those that produce flood, drought, famine, and fire.” [12].

In hermeneutics, generally speaking, emphasis is on the limits of knowledge and knowledge acquisition. Knowledge about how one is to understand oneself and normative knowledge, it is claimed, result from ongoing engagement in social relations. Claims to this kind of knowledge thus remain open to criticism and readjustment. Paul Ricoeur analyzes this situation in terms of political order. Arguing and settling for a common good unites an individual with all other individuals who share these aims. Parts of the individuals’ identity is then defined by these aims. This unity and, accordingly, these personal identities can be challenged, if someone calls into question whether the settled-upon aims suffice to establish the common good. If the following struggle of re-shaping aims is to be successful, it demands that individuals reconsider their identities without having recourse to an overarching position that allows them to judge, once and for all, which changes and amendments to the political order and one’s identity are justified. Individuals who reach consensus on and build political orders accordingly, as well as the political order itself, thus remain inherently vulnerable to struggle and development [13].

In a similar way, Jürgen Habermas describes the formation of personal identity as a process of, on the one hand, incorporating conceptions of the good of a pre-established shared lifeworld. On the other hand, how the individual makes use of these concepts and justifications shapes and changes the shared lifeworld. Building up personal identity thus always stands in need of recognition and is open to critical reevaluation by others and oneself. Habermas claims: “The more the subject becomes individuated, the more he becomes entangled in a densely woven fabric of mutual recognition, that is, of reciprocal exposedness and vulnerability. Unless the subject externalizes himself by participating in interpersonal relations through language, he is unable to form that inner center that is his personal identity. This explains the almost constitutional insecurity and fragility of personal identity – an insecurity that is antecedent to cruder threats to the integrity of life and limb.” [14] This kind of cognitive vulnerability becomes visible in any situation that necessitates a re-shaping of convictions of who one is, and which aims and values are supposed to guide one’s actions. A situation as sketched above in which a patient is confronted with a severe diagnosis obviously fits this description. In addition, this kind of cognitive vulnerability is intimately connected to emotional vulnerability. It was argued above that the process of re-structuring values and preferences and of re-shaping a conception of the good life may be accompanied by anxious insecurity. Now, if one follows Habermas, one may claim in addition that shaping a conception of the good life is a communicative process, or at least a communicable process, the vulnerability of which can be alleviated by recognition. Put simplistically, being met with mistrust and disapproval hurts, being met with respect and approval is enjoyable. Feelings of acceptance and refusal, of inclusion and exclusion, are, then, the emotional aspect of approval or disapproval. What is more, when one enters into evaluative discourse one does not know, yet, how others will react, whether approval or disapproval lie ahead. This cognitive uncertainty goes hand in hand with emotions of anxiety and hope. These emotions constitute, one may claim, the affective side of cognitive vulnerability – in its form of evaluative reasoning –, which is to say that they constitute a specific form of emotional vulnerability.

Following phenomenological and hermeneutic perspectives can help to delineate the various ways in which human existence is characterized by vulnerability. Phenomenological accounts can supply an analysis of physical and corresponding emotional and cognitive vulnerability, and of how these vulnerabilities are experienced. Hermeneutic approaches can supply an account of cognitive vulnerability and, again, its emotional counterpart. Identifying these vulnerabilities can contribute to a full understanding of the situation of healthcare recipients and its ethical implications. What is more, hermeneutic approaches can also help to explain in which ways healthcare providers, too, are marked by vulnerability. Healthcare providers may be able to offer remedies for the physical vulnerability at stake, they may also be able to offer treatment of pathological cases of emotional distress, but if they are asked to assist in re-ordering preferences and assigning value to states of health and disease, they are on the same precarious grounds as the patient is. While physical and emotional vulnerability may appear to be manageable by adhering to expert knowledge, and by devising and administering means accordingly, cognitive vulnerability, when it concerns the re-ordering of one’s preferences and values, calls into question the very ends on which one’s self-understanding and actions are based. It thus unavoidably affects healthcare providers as well as recipients.

### Valuable vulnerability

Pointing out the ethical relevance of cognitive vulnerability strengthens, as was argued, attempts to “symmetrize” the modelling of the healthcare relation. At the same time, doing so introduces a significant shift in the ethical role that the concept of vulnerability is supposed to have. In research ethics and medical ethics, vulnerability is regarded as a state that ought to be overcome. Its regulatory normative role is to signify precarious states of affairs that negatively affect a person’s health status, emotional stability, or cognitive ability to understand and consent to medical procedure. Now, the ethical role of cognitive vulnerability is different. This kind of vulnerability brings with it exposedness, but it is at the same time the prerequisite of developing a self-understanding that comprises an idea of the convictions and values that one aims to conform to. If one were to avoid exposedness by reducing evaluative cognitive vulnerability, this would amount to forgoing the task to become an agent who is capable of explaining and justifying his actions. With respect to evaluative cognitive vulnerability, then, the ethical demand is not to overcome, but to establish and maintain vulnerability. In addition, if evaluative cognitive vulnerability corresponds to certain forms of emotional vulnerabilities, these, too, would have to be maintained rather than abolished.

A similar point is often made regarding vulnerabilities in relationships such as friendship and love [15]. Entering into a close personal relation puts one at risk. It means to offer one’s liking and friendship without guarantee that they will be welcomed and returned. Even in long term relationships, this risk remains present simply because the other one is an *other*. Relations necessarily involve trust, and trust can always be betrayed. In this sense, being in relations entails emotional vulnerability. At the same time, maintaining close personal relationships is experienced as rewarding, supports self-confidence, and can be the source of honest feedback, to name but a few of the positive aspects. Undoubtedly, relationships can acquire pathological patterns and thus can come to impose vulnerabilities on one or more of the persons involved that are to be viewed on par with any other of those vulnerabilities that are to be ameliorated. Still, since a certain degree of emotional vulnerability in close personal relationships is a prerequisite of having these relationships, it does not make ethical sense to aim to reduce this degree of vulnerability. Perhaps, but this may be an issue for debate, it is possible to interpret emotional vulnerability in personal relations as another form of those emotional vulnerabilities that are tied to evaluative cognitive vulnerability. After all, close relations may be regarded as prime realms for exposing, disputing, developing and acknowledging convictions and values and conceptions of a good life [16].

## Conclusions

Vulnerability permeates the relation of healthcare recipients and providers and it points to many of the ethically relevant aspects of this relation. Vulnerability comprises physical, emotional, and cognitive phenomena and thus allows to identify ethical tasks of providing assistance for patients besides and in addition to medical assistance. What is more, the concept of vulnerability highlights the intimate connection between physical, emotional, and cognitive phenomena and thus encourages a comprehensive understanding of the patient’s situation. Philosophical approaches to vulnerability can help to come closer to a systematization of forms and degrees of physical, emotional, and cognitive vulnerability, and their interconnections.

An inherent danger of turning vulnerability into a core concept of medical ethics is to picture the healthcare recipient as a purely passive person in need and the provider as a person able to provide all the assistance needed. As was argued, including evaluative cognitive vulnerability can help to overcome this danger. Evaluative cognitive vulnerability affects both healthcare recipients and providers. While physical and emotional vulnerability may appear to be manageable by adhering to expert knowledge, and thus possibly reinforce the idea of the recipient as needy and passive and the provider as powerful and active, evaluative cognitive vulnerability calls into question the very ends on which one’s self-understanding and actions are based. Confronted with questions of this kind, the healthcare provider does not, qua being a care expert, have better resources to come up with convincing answers than the care recipient. Pointing out the ethical relevance of cognitive vulnerability helps to “symmetrize” the modelling of the healthcare relation. An ethic based on vulnerability thus joins approaches and concepts such as shared decision-making, empowerment, and health promotion. It does so in a specific way, though, namely by interpreting the symmetry between patient and physician as a symmetry of shared vulnerability.

Finally, this introduces a significant shift in the ethical role that the concept of vulnerability is supposed to have. While vulnerability in medical ethics usually is thought of as a state of affair that needs to be ameliorated or overcome, evaluative cognitive vulnerability and its emotional correlates are to be maintained. Further analyzing and systematizing forms and degrees of vulnerability thus also comprises the task to distinguish between amounts and types of vulnerability that can count as prerequisites of being an agent who develops and holds normative convictions, and amounts and types of vulnerability that all too easily can result in harm or have already turned into harm.
